# Hemolytic Properties of Fine Particulate Matter (PM_2.5_) in In Vitro Systems

**DOI:** 10.3390/toxics12040246

**Published:** 2024-03-27

**Authors:** Jiahui Bai, Mengyuan Zhang, Longyi Shao, Timothy P. Jones, Xiaolei Feng, Man Huang, Kelly A. BéruBé

**Affiliations:** 1 State Key Laboratory of Coal Resources and Safe Mining, College of Geoscience and Surveying Engineering, China University of Mining & Technology, Beijing 100083, China; 15010604001@163.com (J.B.); feng12xiaolei@163.com (X.F.); huangman08@sina.com (M.H.); 2 Postdoctoral Research Base, School of Resource and Environment, Henan Institute of Science and Technology, Xinxiang 453000, China; 3 School of Earth and Environmental Sciences, Cardiff University, Museum Avenue, Cardiff CF10 3YE, UK; jonestp@cardiff.ac.uk; 4 School of Biosciences, Cardiff University, Museum Avenue, Cardiff CF10 3AX, UK; berube@cardiff.ac.uk

**Keywords:** erythrocyte, health risk, hemolysis, plasmid scission assay, PM_2.5_, toxicity index

## Abstract

Epidemiological studies have suggested that inhalation exposure to particulate matter (PM) air pollution, especially fine particles (i.e., PM_2.5_ (PM with an aerodynamic diameter of 2.5 microns or less)), is causally associated with cardiovascular health risks. To explore the toxicological mechanisms behind the observed adverse health effects, the hemolytic activity of PM_2.5_ samples collected during different pollution levels in Beijing was evaluated. The results demonstrated that the hemolysis of PM_2.5_ ranged from 1.98% to 7.75% and demonstrated a clear dose–response relationship. The exposure toxicity index (TI) is proposed to represent the toxicity potential of PM_2.5_, which is calculated by the hemolysis percentage of erythrocytes (red blood cells, RBC) multiplied by the mass concentration of PM_2.5_. In a pollution episode, as the mass concentration increases, TI first increases and then decreases, that is, TI (low pollution levels) < TI (heavy pollution levels) < TI (medium pollution levels). In order to verify the feasibility of the hemolysis method for PM toxicity detection, the hemolytic properties of PM_2.5_ were compared with the plasmid scission assay (PSA). The hemolysis results had a significant positive correlation with the DNA damage percentages, indicating that the hemolysis assay is feasible for the detection of PM_2.5_ toxicity, thus providing more corroborating information regarding the risk to human cardiovascular health.

## 1. Introduction

In recent years, due to the rapid development of industry and the increasing demand for energy such as coal and petroleum, China has been in a period of frequent occurrences of air pollution incidents with PM_2.5_ (airborne particulate matter with an aerodynamic diameter of 2.5 microns or less) as the dominant pollutant. The severe haze episodes occurred frequently during the heating seasons (autumn and winter), especially in Beijing [1,2]. At present, domestic and foreign research has investigated the sources of atmospheric PM_2.5_ from the aspects of ground dust and biomass combustion, its transformation, and physical and chemical properties [3]. Many studies have inferred that the impact of inhaling atmospheric particulates on human health is detrimental [4,5,6,7]. In 2013, the International Agency for Research on Cancer defined air pollutants, including PM_2.5_, as Class I carcinogens, that is, “identified human carcinogens”, which has aroused the public’s concern about air pollutants and health [8].

As an important part of air pollution, PM_2.5_ in the atmosphere has caused widespread concern for its impact on human health [9,10]. There is a large body of epidemiological evidence stating that a significant positive correlation exists between long-term exposure to airborne PM and increased health risk and mortality from cardiovascular diseases [11,12,13]. The composition of PM_2.5_ is very complex and diverse, containing a range of toxic substances such as heavy metals, polycyclic aromatic hydrocarbons (PAHs), sulfates, bacteria, and viruses [14,15]. The sub-micron toxic substances can enter the respiratory system and penetrate the intima or enter the capillary lumen via endocytosis [16]. Shimada et al. found that PM_2.5_ could enter blood circulation in a short time through the “interstitial permeation pathway” induced by the air–blood barrier. PM_2.5_, which enters into circulation, can interact directly with blood cells and vascular tissues, affecting the quality of blood cells, body tissues, and organs [17,18,19]. In spite of this, the biological plausibility of the adverse health effects of ambient particles remains unclear. Many methods have been used to assess the toxicity of particles, incorporating both in vivo [20,21] and in vitro methods [22,23,24]. Due to complicated experimental processes, long test periods, and high costs of in vivo methods, researchers are now utilizing in vitro protocols for rapid toxicological analysis of atmospheric PM, such as the hemolysis assay [25,26], plasmid scission assay (PSA) [27], and apoptosis assay [22,23].

Hemolysis assay is a convenient and rapid in vitro toxicological test that determines the toxicity of PM_2.5_ by evaluating the hemolysis of a suspension of red blood cells (RBCs) in contact with PM_2.5_ [28,29,30]. Due to its small particle size and large specific surface area, PM_2.5_ adsorbs a large number of oxidizing substances [31,32]. These oxidizing substances can generate reactive oxygen species (ROS) in aqueous solutions [33]. ROS is cytotoxic, and when in contact with the RBC membrane, it will increase its fluidity and permeability and induce lipid peroxidation, culminating in hemolytic activity (i.e., destruction of RBC membranes) [34]. Destruction of RBCs can lead to adverse effects such as anemia, jaundice, and other pathological conditions [34]. Since PM_2.5_ cannot be completely removed by the human body in a short time, a small part will accumulate in the lungs or be transferred to regional lymph nodes [35]. It can be inferred that if people are exposed to the atmospheric environment polluted by PM_2.5_ for extended periods of time, the toxic effects of particles will become a significant health risk.

In this study, outdoor PM_2.5_ samples were collected in Beijing under different pollution levels, and the hemolysis test was performed on PM_2.5_ to reveal the toxicology of the particles. PSA was also undertaken on the PM_2.5_ samples, and the results were compared with the hemolysis results to verify the feasibility of the hemolysis test. The combination of two methods can provide more corroborating information regarding the risk to human cardiovascular health.

## 2. Sampling and Experiments

### 2.1. Sample Collection

The sampling site (116°20′45.6″ E, 39°59′37.1″ N) was located at the China University of Mining and Technology (Beijing) in northwestern Beijing. The sampling point was 17.8 m above the ground, approximately 1 km from Beijing’s north 4th Ring Road. This collection site is part of a typical university campus and residential area in Beijing, with no large, heavy industrial pollution sources.

A TSP-PM_10_-PM_2.5_ Sampler (KB-120E, Qingdao, China) and quartz microfiber filters (90 mm, Whatman, China) were used to collect PM_2.5_ at a flow rate of 100 L/min. A Pocket Weather Tracker (Kestrel 5500 Weather LiNK, Minneapolis, MN, USA) was used to record meteorological data during sampling. Before sampling, the quartz fiber filters were heated at 450 °C for 4 h in a muffle furnace (ZK-6XY-1400, Beijing, China) and placed in a constant temperature and humidity chamber (Hitachi, Japan; temperature: 20 ± 5 °C; relative humidity: 45 ± 5%).

The mass concentrations of PM_2.5_ were obtained using the gravimetric method. The fiber filters were weighed using an electronic balance (Sartorius CP225D, Göttingen, Germany) with an accuracy of 0.01 mg. The formula calculating the mass concentration, as defined by Feng et al. (2022), was employed [36]. According to the mass concentration of PM_2.5_, the pollution degree was divided into low (0–74 μg·m^−3^), medium (75–150 μg·m^−3^), and heavy pollution levels (>150 μg·m^−3^; Table 1). Sample G is a special case where the rain resulted in an average PM_2.5_ concentration below 150 μg·m^−3^. However, data from nearby monitoring sites from Beijing Municipal Ecology and Environment Bureau showed that the average concentration during the first 11 h of the sampling period exceeded 155 μg·m^−3^. Considering the overall pollution situation, we categorized sample G as having a heavy pollution level.

### 2.2. Preparation of PM_2.5_ Suspensions

The filters (including the blank filter) were cut into 5 mm^2^ and put into a 15 mL centrifuge tube (Corning, NY, USA). A measured amount of phosphate-buffered saline (PBS, Sigma, Dorset, UK) was added to the centrifuge tube to make a particle dosage of 100 μg/mL. The centrifugal tube was placed into a platform shaker (VORTEX-GENIE2, Scientific Industries, New York City, NY, USA) and mixed for 20 h to obtain a PM_2.5_ suspension.

### 2.3. Hemolysis Assay

The cytoplasm of erythrocytes is rich in hemoglobin. When RBC ruptures (i.e., hemolysis), the hemoglobin is released into the plasma, changing the plasma from being relatively colorless to having a red tint. The percentage of hemolysis can be measured by separating the plasma from the RBCs and analyzing the amount of cell-free hemoglobin using a spectrophotometer [28]. The degree of hemolysis is reflective of the toxicity of the PM samples.

The rabbit blood samples (Xinglong Laboratory Animal Breeding Plant, China) were centrifuged at 1500 rotations per minute (rpm) (Martin Christ, Osterode, Germany) at 4 °C for 10 min; the supernatant and a thin layer of platelets were removed from the blood and discarded. The blood samples were re-suspended with PBS to make them back up to 7 mL; this was repeated 2–3 times. The final working solution was 2% RBC suspension diluted with PBS.

The PM_2.5_ samples were suspended in PBS and diluted into 5 doses: 1000 μg/mL, 500 μg/mL, 400 μg/mL, 200 μg/mL, 100 μg/mL), 2 parallel samples per dose. The PM_2.5_ suspensions (125 μL) were added to the prepared blood (125 μL) in a 96-well (Cole Palmer, Cambridgeshire, UK) plate, which was then sealed with adhesive plastic and placed on a platform shaker (HX-3000, YOUNING, Jinan, China) for 60 min at room temperature. The plate was then centrifuged at 200 rpm for 5 min, after which 200 μL of supernatant was removed and placed into a corresponding well of a new 96-well plate. The negative control contained PBS, and the positive control contained sodium dodecyl sulfate (SDS, 1.3 g/L) mixed with the prepared blood. The plate containing the supernatant had its optical density read using the Tecan Infinite^®^ 200 PRO plate reader at 540 nm. Each sample was measured 3 times, and the results were averaged. The absorbance readings in optical density from the plate reader were converted into percentage of hemolysis with the following formula:

% hemolysis = [(sample OD − negative control OD)/(positive control OD − negative control OD) × 100]

### 2.4. Plasmid Scission Assay

The PSA is an in vitro method for quantitatively measuring the oxidative damage capacity of ROS to plasmid DNA. Its basic principle is that free radicals carried on the surface of particles will cause oxidative damage to supercoiled DNA, initially causing supercoiled DNA to relax, and further damage will induce DNA linearization [3]. The relative electrophoretic mobility of supercoiled, relaxed, and linearized DNA in the gel analysis system (Synoptics Ltd., Cambridge, UK) was used to calculate the percentages of the three forms of plasmid DNA. The total percentages of the relaxed and linearized DNA were taken as the oxidative damage capacity. The detailed experimental procedures were conducted as described in Feng et al. (2022) [36].

Different doses of PM_2.5_ suspensions (i.e., 1000 μg/mL, 500 μg/mL, 400 μg/mL, 200 μg/mL, 100 μg/mL, 50 μg/mL) were prepared to determine the DNA damage rate. A total of 41 μL of the sample supernatant and 2 μL of the plasmid X174-RF DNA (Promega, Madison, WI, USA) was added to a 1.5 mL centrifuge tube. This was oscillated horizontally for 6 h to ensure a good mixing (HX-3000, YOUNING, Jinan, China). Agarose (molecular biology grade; Sigma-Aldrich, Beijing, China) was dissolved in 100 X Tris/Borate/EDTA (TBE) buffer solution (Thermo Fisher Scientific, Waltham, MA, USA), and the solution was heated to transparency. Ten microliters of ethidium bromide (EB; Sigma-Aldrich, China) was added to the agarose solution when cooled to 78 °C, and this formed a gel on the electrophoresis plate (DYCP34C; LIUYI, Beijing, China).

Seven microliters of bromophenol blue stain (Sigma-Aldrich, China) were added to the mixture of the sample supernatant, and DNA was injected into the solidified gel wells. Each gel well was injected with 20 μL of the final solution. The electrophoresis apparatus (DYY-6C, LIUYI, China) was operated at 30 V for 16 h at room temperature.

The variation in DNA morphologies was observed and quantified by the UV gel imaging system (ChemiDoc, Biored, Shanghai, China) and the Syngene Genetools software (version 4.0; Syngene, Cambridgeshire, UK).

### 2.5. Toxicity Index (TI)

The toxicity index (TI) of PM_2.5_ refers to the degree of damage to erythrocyte hemolysis exhibited by the PM mass per unit volume of air; that is, the greater the exposure toxicity index, the higher the health risk for human exposure, which is:

TI = ρ × Z(1)

In the formula, TI is the toxicity index of PM_2.5_ exposure, ρ is the mass concentration of PM_2.5_ (μg/m^3^), and Z is the hemolysis percentage at 500 μg/mL PM_2.5_ dose.

### 2.6. Quality Assurance/Quality Control

Appropriate blanks and parallel samples were analyzed with each set of samples. The blank control in the hemolysis assay was PBS, and 3 parallel samples were set for each concentration. If its hemolysis was not higher than 0.5%, the result was considered meaningful. The blank control in the PSA was ultrapure water, and 2 parallel samples were set for each concentration. If the DNA damage rate was not higher than 1%, the result was considered meaningful.

### 2.7. Statistical Analysis

All analyses were performed using SPSS Statistics (version 26.0) and Excel 2010 software for Windows.

## 3. Results

### 3.1. Hemolysis of PM_2.5_ during a Pollution Episode

The hemolysis values are shown in Table 2. Hemolysis ranged from 1.98% to 7.75%, with sample G having the lowest value, with 1.98% at a dose of 150 μg/mL, and sample B having the highest value, with 7.75% at a dose of 1000 μg/mL. The hemolysis rate of samples collected at heavy pollution levels was significantly lower than that of low pollution levels, which indicated that the toxicity of PM_2.5_ was independent of the level of pollution. This is consistent with a previous study, which concluded that the unit toxicity of PM_2.5_ is related to its chemical composition (such as the content of heavy metal elements), regardless of the weather conditions [27].

### 3.2. DNA Damage of PM_2.5_ during a Pollution Episode

PM_2.5_ samples from low, medium, and heavy pollution levels were analyzed using PSA to determine the DNA damage rate across a range of doses (i.e., 50, 100, 200, 400, 500, and 1000 μg/mL; Table 3 and Figure S1). DNA damage rate ranged from 17.06% to 39.19%, with sample G having the lowest value, with 17.06% at a dose of 50 μg/mL, and sample D having the highest value, with 39.19% at a dose of 1000 μg/mL. The DNA damage rate of samples collected at heavy pollution levels is obviously lower than that of low pollution levels.

## 4. Discussion

### 4.1. Comparison of Hemolysis Assay and Plasmid Scission Assay

**Figure 1:**
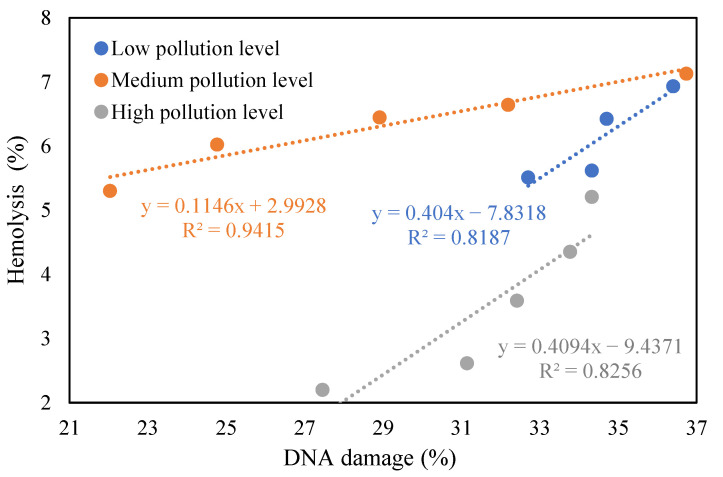
Comparative analysis of hemolysis rate and DNA damage rate under different pollution levels.

PSA has been proven in a number of studies to accurately reflect the toxicity of PM [3,36]. Therefore, this study was conducted to verify the feasibility of the hemolysis method for PM toxicity detection using the DNA damage rate. A comparison of the results of the PSA and hemolysis assay is shown in Figure 1. The results showed that for the same PM_2.5_ sample of the same pollution process, the results obtained by two different toxicological methods showed the same trend, i.e., medium pollution level > low pollution level > high pollution level. The results of a linear regression line fitting showed that the R^2^ values were in the range of 0.82–0.94, close to 1, indicating a significant positive correlation between DNA damage and hemolysis rates. This positive correlation demonstrated that the hemolysis assay is feasible for the detection of PM_2.5_ toxicity, and the experimental results obtained were of scientific significance.

### 4.2. Dose–Response Relationship between Hemolysis and PM_2.5_

**Figure 2:**
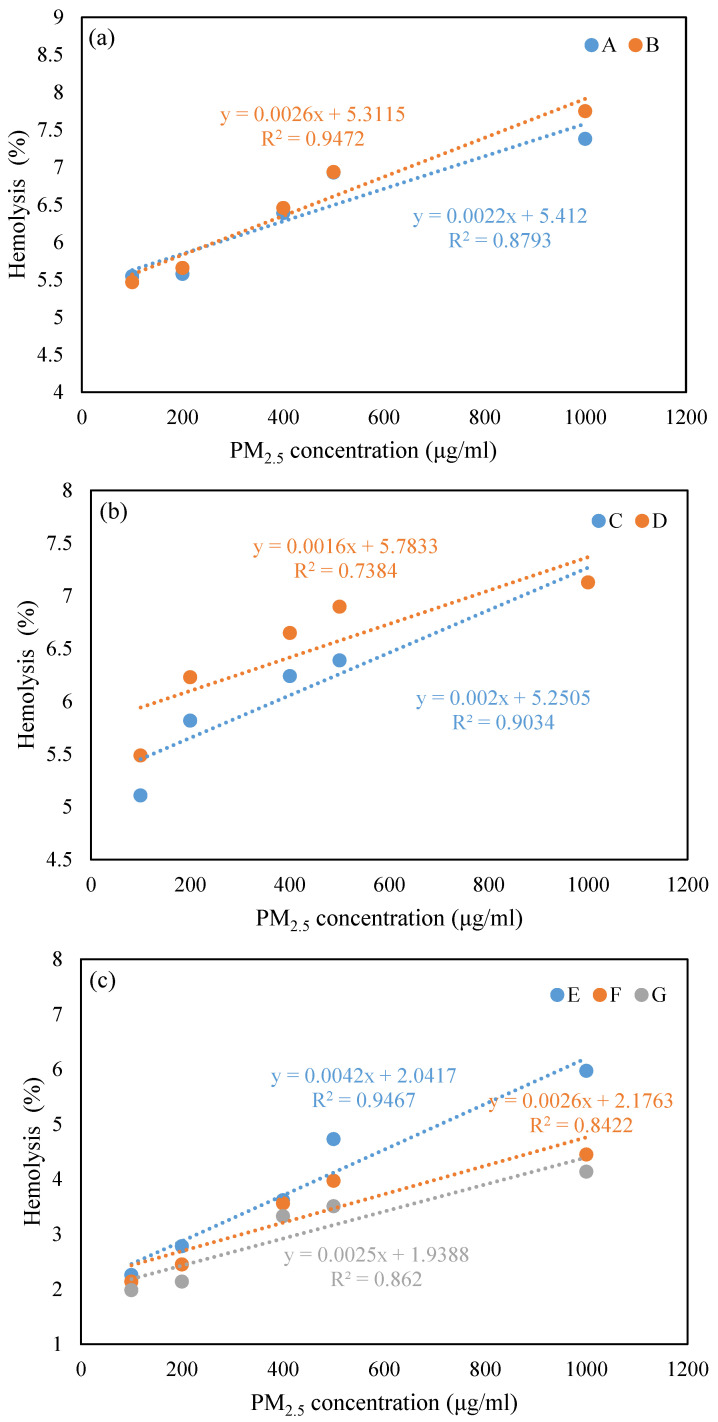
Correlation analysis between PM_2.5_ concentration and hemolysis: (**a**) low PM_2.5_ pollution level; (**b**) medium PM_2.5_ pollution level; (**c**) high PM_2.5_ pollution level.

The results of linear regression line fitting (Figure 2) showed that hemolysis was positively correlated with the doses of PM_2.5_ (R^2^ = 0.74–0.95), which demonstrated that the hemolysis rate increased with the concentration of PM_2.5_. A similar study in the Mexico–US border region reflected the same consistency in an increasing trend in the RBC hemolysis rate and PM_2.5_ concentrations [37]. Similar results have been reported in an in vivo study: oxidative and metabolic damage occurred in a dose-dependent manner with increasing particle concentration [16]. Studies show that PM_2.5_ will not be completely removed by the human body in a short time, and a small part will accumulate in the lungs or be transferred to regional lymph nodes [35]. Therefore, due to the dose–response relationship between hemolysis and PM_2.5_, the hemolytic effect induced by PM_2.5_ will increase the health risk to humans with the accumulation of time.

The TD_10_ (toxic dose causing 10% hemolysis) of PM_2.5_ samples was calculated to characterize the toxicity of PM_2.5_ samples, and a lower TD_10_ means a higher toxicity. PM_2.5_ in Beijing during a pollution episode had a TD_10_ value ranging from 1803.27 μg/mL to 3224.48 μg/mL, with an average of 2432.48 μg/mL. The TD_10_ of PM_2.5_ in this study was significantly higher than that in a previous study in Tehran, which concluded that the mean value TD_10_ of PM_10_ during a dust storm was 150 μg/mL and the mean value TD_10_ of PM_10_ during a weather inversion was 220 μg/mL [26]. This indicated that thanks to the implementation of a series of pollution prevention and control measures from 2013 to 2017, the source structure of PM_2.5_ in Beijing has changed, and its chemical composition has also changed, resulting in a decrease in toxicity.

### 4.3. Exposure Risk of PM_2.5_ during a Pollution Episode

Although the toxicity per unit of PM in heavily polluted weather was low, the overall exposure toxicity caused by the large concentration of PM was higher than that of unpolluted weather. Therefore, the PM_2.5_ exposure toxicity index (TI) was introduced to characterize the overall exposure health risks of people under different pollution levels (USEPA 2014).

**Figure 3:**
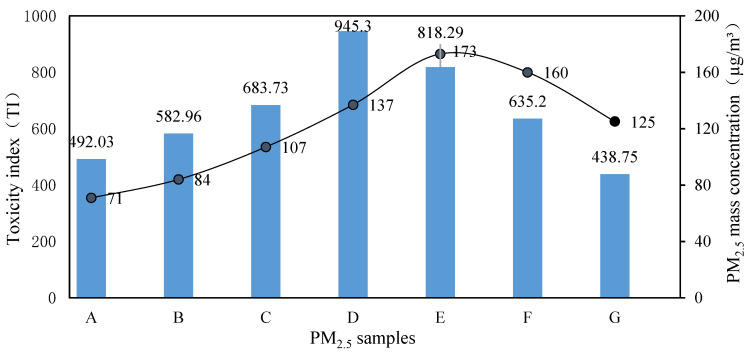
Toxicity index (TI) of PM_2.5_ samples at different mass concentrations.

As shown in Table 4, the TI of PM_2.5_ collected during a medium pollution level (average value was 815) was much higher than that during a low pollution level (average value was 537) and a heavy pollution level (average value was 631). In a pollution episode, as the mass concentration increased, the toxicity index first increased and then decreased (Figure 3), that is, TI (low pollution levels) < TI (heavy pollution levels) < TI (medium pollution levels). Previous studies have shown that there is no correlation between the concentration of PM_2.5_ during the sampling period and its unit toxicity [36]. The toxicity of PM_2.5_ is only related to its composition and, furthermore, to the source of PM_2.5_ [28]. Therefore, two factors should be considered together when assessing the exposure risk of PM_2.5_: (1) the concentration of PM_2.5_ and (2) the unit toxicity of PM_2.5_. A high toxicity with a low concentration and a low toxicity with a high concentration are not necessarily indicative of high exposure risk; rather, it is the situation where both are at medium levels, which we are most likely to overlook, that may pose a greater risk to population health. It implies that in the future, not only heavy pollution but also medium pollution weather should be included as a key concern when making environmental regulations.

Although this study confirms the hemolysis effect of PM_2.5_, the current work still has certain limitations. More samples of the pollution process need to be analyzed to make the conclusion more convincing. Nevertheless, our findings revealed the dose–response relationship between hemolysis and PM_2.5_ and quantified the exposure risk of the PM_2.5_ hemolysis effect during pollution. It provides a warning for the cardiovascular health of people living in severely air-polluted areas.

## 5. Conclusions

In this investigation, we tested the feasibility of using the hemolysis assay as a viable test to assess the toxicity of PM samples. In a pollution episode, the hemolysis of PM_2.5_ ranged from 1.98% to 7.75% and demonstrated a clear dose–response relationship. PSA was utilized to verify the results obtained by the hemolysis assay. The DNA damage rates obtained by PSA had a significant positive correlation with the results from the hemolysis assay, indicating that the hemolysis assay is feasible for the detection of PM_2.5_ toxicity. The toxicity index (TI) was utilized to estimate the overall risks to human health (i.e., respiratory toxicity) following PM exposures at different pollution levels. In a pollution episode, as the mass concentration increased, the toxicity index first increased and then decreased, that is, TI of low pollution levels < TI of heavy pollution levels < TI of medium pollution levels.

## Figures and Tables

**Table 1:** Sampling information.

Sample Number	Sampling Date	SamplingTime	Sampling Duration	Pollution Level	Weather	PM_2.5_ Mass Concentration (μg·m^−3^)
A	12 October 2018	18:30–7:30	13 h	Low	Sunny	71
B	13 October 2018	8:30–16:30	8 h	Low	Sunny	84
C	13 October 2018	18:30–7:30	13 h	Medium	Sunny	107
D	14 October 2018	8:30–16:30	8 h	Medium	Sunny	137
E	14 October 2018	18:30–7:30	13 h	Heavy	Sunny	173
F	15 October 2018	8:30–16:30	8 h	Heavy	Sunny	160
G	15 October 2018	18:30–7:30	13 h	Heavy	Rain	125

**Table 2:** Quantification of hemolysis induced by PM_2.5_ (%).

Sample Number	Dose Concentration (μg /mL)				
	100	200	400	500	1000
A	5.55 ± 0.97	5.58 ± 0.51	6.39 ± 0.54	6.93 ± 0.64	7.38 ± 0.20
B	5.47 ± 0.82	5.66 ± 0.67	6.46 ± 0.21	6.94 ± 0.49	7.75 ± 0.51
C	5.11 ± 0.03	5.82 ± 0.22	6.24 ± 0.42	6.39 ± 0.62	7.13 ± 0.75
D	5.49 ± 0.38	6.23 ± 0.38	6.65 ± 0.04	6.9 ± 0.96	7.13 ± 0.80
E	2.26 ± 0.68	2.78 ± 0.20	3.62 ± 0.57	4.73 ± 0.62	5.97 ± 0.93
F	2.14 ± 0.34	2.45 ± 0.14	3.56 ± 0.48	3.97 ± 0.83	4.45 ± 0.64
G	1.98 ± 0.59	2.14 ± 0.90	3.33 ± 0.96	3.51 ± 0.25	4.14 ± 0.86

**Table 3:** Plasmid DNA damage rate induced by PM_2.5_ (%).

Sample Number	Dose Concentration (μg/mL)					
	50	100	200	400	500	1000
A	28.74	31.00	33.48	33.64	34.50	-
B	21.82	34.41	35.18	35.77	38.31	-
C	-	21.40	21.59	27.51	32.80	34.29
D	-	22.68	27.94	30.32	31.58	39.19
E	-	28.21	31.81	33.13	34.07	35.01
F	-	26.7	30.48	31.71	33.48	33.64
G	17.06	18.73	20.24	22.83	24.04	-

Note: “-” means “not analyzed”.

**Table 4:** Hemolysis rate and toxicity index (TI) at different PM_2.5_ concentrations.

Sample Number	Mass Concentration (μg/m^3^)	The Hemolysis of the Sample at a Dosage of 500 μg/mL for Samples	Toxicity Index (TI)
A	71	6.36	492.03
B	84	6.48	582.96
C	107	6.94	683.73
D	137	6.9	945.3
E	173	4.73	818.29
F	160	4.02	635.2
G	125	3.97	438.75

## Data Availability

Data are contained within the article and Supplementary Materials.

## Supplementary Materials

The following supporting information can be downloaded at: https://www.mdpi.com/article/10.3390/toxics12040246/s1, Figure S1: chromatograms of electrophoretic by PSA.
