# Supplementary figures and images for: Hemolytic Properties of Fine Particulate Matter (PM2.5) in In Vitro Systems
Source: Toxics. 2024 Mar 27;12(4):246. doi: 10.3390/toxics12040246 (PMC11054038; doi:10.3390/toxics12040246)

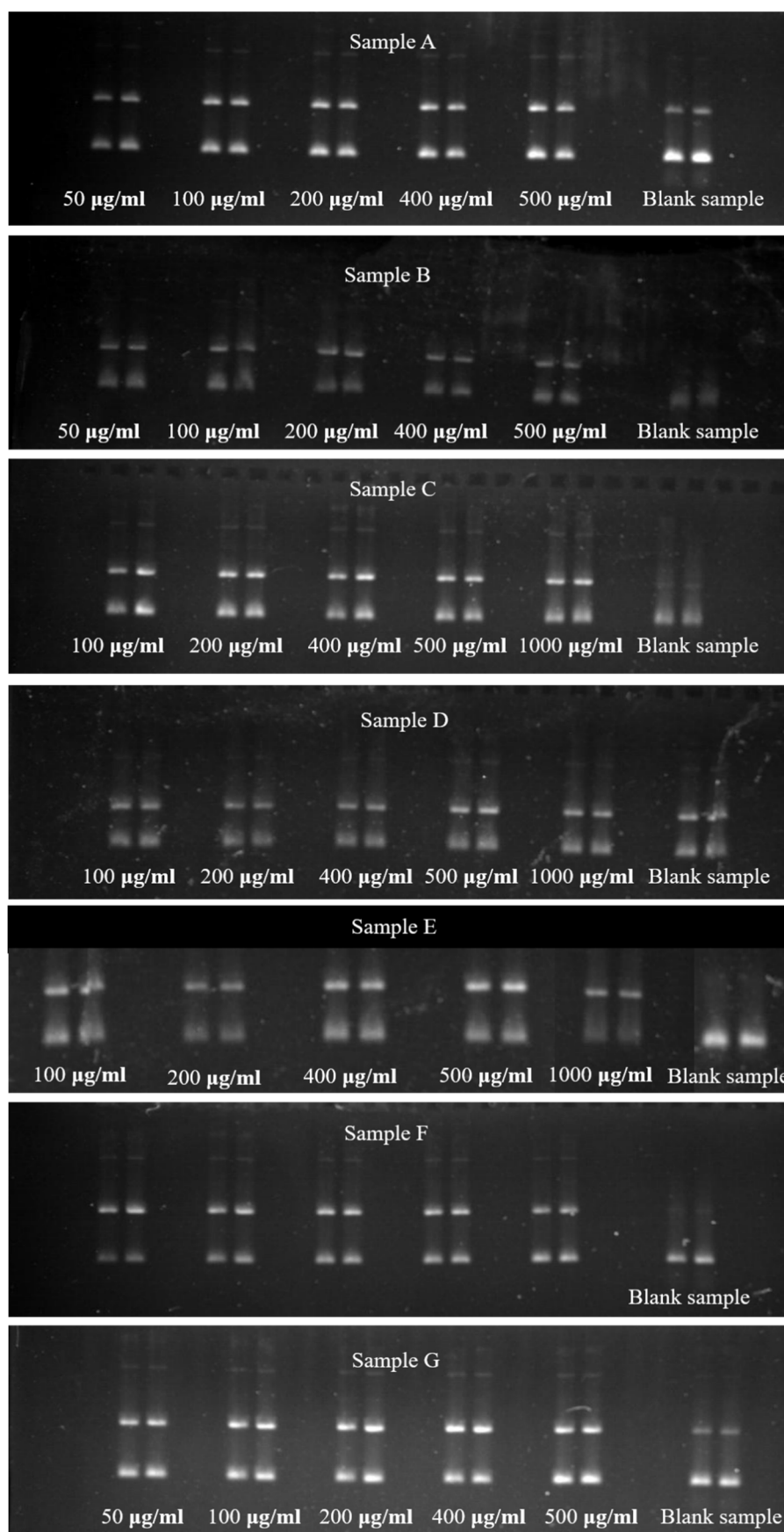

Figure S1 Chromatograms of electrophoretic by PSA

Supplement: Supplementary file 1 [file toxics-12-00246-s001.zip › toxics-2892741-supplementary (3).pdf]
